# Gene fusions are frequent in ACTH-secreting neuroendocrine neoplasms of the pancreas, but not in their non-pancreatic counterparts

**DOI:** 10.1007/s00428-022-03484-4

**Published:** 2023-01-24

**Authors:** Abbas Agaimy, Atsuko Kasajima, Robert Stoehr, Florian Haller, Christoph Schubart, Lars Tögel, Nicole Pfarr, Alexander von Werder, Marianne E. Pavel, Fausto Sessa, Silvia Uccella, Stefano La Rosa, Günter Klöppel

**Affiliations:** 1 Institute of Pathology, University Hospital Erlangen, Friedrich Alexander University of Erlangen-Nuremberg & Comprehensive Cancer Center, European Metropolitan Area Erlangen-Nuremberg (CCC ER-EMN), Erlangen, Germany; 2 Institute of Pathology, Technical University Munich, Munich, Germany; 3 Department of Internal Medicine 2, Technical University Munich, Munich, Germany; 4 Department of Medicine 1, Division of Endocrinology, Comprehensive Cancer Center, Erlangen University Hospital, European Metropolitan Area Erlangen-Nuremberg (CCC ER-EMN), Friedrich Alexander University of Erlangen-Nuremberg, Erlangen, Germany; 5 Unit of Pathology, Department of Medicine and Surgery, University of Insubria, Varese, Italy; 6 Humanitas University, Pieve Emanuele, Milan, Italy; 7 Humanitas Research Hospital, Rozzano, Milan Italy

**Keywords:** ACTH; Neuroendocrine tumor; NET, ectopic Cushing; EWSR1, BEND2 fusion; KMT2A, BCOR fusion

## Abstract

Ectopic Cushing syndrome is a rare clinical disorder resulting from excessive adrenocorticotrophic hormone (ACTH) produced by non-pituitary neoplasms, mainly neuroendocrine neoplasms (NENs) of the lung, pancreas, and gastrointestinal tract, and other less common sites. The genetic background of ACTH-producing NENs has not been well studied. Inspired by an index case of ACTH-producing pancreatic NEN carrying a gene fusion, we postulated that ACTH-producing NENs might be enriched for gene fusions. We herein examined 21 ACTH-secreting NENs of the pancreas (10), lung (9), thymus (1), and kidney (1) using targeted RNA sequencing. The tumors were classified according to the most recent WHO classification as NET-G1/typical carcinoid (*n* = 4), NETG-2/atypical carcinoid (*n* = 14), and NET-G3 (*n* = 3). Overall, targeted RNA sequencing was successful in 11 cases (4 of 10 pancreatic tumors, 5 of 9 pulmonary tumors, and in the one renal and one thymic tumor). All four successfully tested pancreatic tumors revealed a gene fusion: two had a *EWSR1::BEND2* and one case each had a *KMT2A::BCOR* and a *TFG::ADGRG7* fusion, respectively. *EWSR1* rearrangements were confirmed in both tumors with a *EWSR1::BEND2* by FISH. Gene fusions were mutually exclusive with *ATRX*, *DAXX*, and *MEN1* mutations (the most frequently mutated genes in NETs) in all four cases. Using RNA-based variant assessment (*n* = 16) or via the TSO500 panel (*n* = 5), no pathogenic BCOR mutations were detected in any of the cases. Taken together, gene fusions were detected in 4/4 (100%) pancreatic versus 0/7 (0%) non-pancreatic tumors, respectively. These results suggest a potential role for gene fusions in triggering the ACTH production in pancreatic NENs presenting with ectopic Cushing syndrome. While the exact mechanisms responsible for the ectopic ACTH secretion are beyond the scope of this study, overexpressed fusion proteins might be involved in promoter-mediated overexpression of pre-ACTH precursors in analogy to the mechanisms postulated for *EWSR1::CREB1*-mediated paraneoplastic phenomena in certain mesenchymal neoplasms. The genetic background of the ACTH-producing non-pancreatic NENs remains to be further studied.

## Introduction

Ectopic Cushing syndrome is a diagnostically and therapeutically challenging rare clinical disorder resulting from chronic exposure to excessive adrenocorticotrophic hormone (ACTH) secreted by neoplasms of non-pituitary origin [1]. These ACTH-producing tumors are generally divided into two main categories: highly aggressive poorly differentiated carcinomas (small cell lung cancer being their prototype), and well-differentiated neuroendocrine tumors (NET) of diverse organs [1, 2]. Among the latter, bronchopulmonary carcinoids [3], gastroenteropancreatic NETs [4], pheochromocytoma/paraganglioma [5], and much less frequently, thymic NET [6], medullary thyroid carcinoma [7], NET of unknown origin [8], and rare anecdotal sites have been well documented as sources of ectopic Cushing syndrome. Moreover, rare non-neuroendocrine neoplasms may be associated with ectopic Cushing syndrome that resolves after tumor resection [4, 9, 10].

Pancreatic ACTH-producing neuroendocrine neoplasms (NENs) are rare with < 150 cases reported to date, mostly as single case reports or rare case series [4, 11, 12]. They are generally more aggressive with an overall 5-year survival of 35%, compared to 72–97% for most other hormone-secreting NETs [4].

To date, the pathogenetic mechanisms underlying the ectopic ACTH production by certain NENs and their genotypes remain obscure. Several theories have been postulated to explain the ectopic hormone production, but none could be proven. Based on an index pancreatic NEN that we have encountered to harbor a *EWSR1::BEND2* gene fusion detected by targeted RNA sequencing, we examined a total of 21 ACTH-producing NENs from pancreatic, pulmonary, thymic, and renal origins to test the hypothesis that the *EWSR1::BEND2* fusion or other alternate gene fusions might be enriched among these rare tumors.

## Material and methods

The cases were identified from the consultation files of the authors and from the routine pathology files of the University Hospital Erlangen, Germany. The tissue specimens were fixed in formalin and processed routinely for histopathology. Due to the consultation nature of the cases, immunohistochemistry (IHC) was performed in different laboratories and the stains applied varied from case to case, based on tissue availability and initial differential diagnostic considerations (details of the staining protocols and antibody sources are available upon request).

A total of 21 ACTH-producing NENs from the pancreas (*n* = 10), the lung (*n* = 9), the kidney (*n* = 1), and the mediastinum/thymus (*n* = 1) have been collected. All tumors have been diagnosed, subtyped, and graded according to the most recent World Health Organization (WHO) classification of neuroendocrine neoplasms for the respective organ [12, 13]. ACTH production by the tumor cells was verified in all 21 cases, both clinically (manifested Cushing syndrome or elevated serum ACTH level) and histologically via ACTH immunohistochemistry. Based on a recent description of a *BCOR* mutation in a case of typical lung carcinoid with ectopic ACTH production [14] and the presence of a BCOR fusion in one of our cases, we also performed BCOR IHC on all cases.

### Next-generation sequencing

RNA was isolated from formalin-fixed paraffin embedded (FFPE) tissue sections using RNeasy FFPE Kit of Qiagen (Hilden, Germany) and quantified spectrophotometrically using NanoDrop-1000 (Waltham, USA). Molecular analysis was performed using the TruSight RNA Fusion panel (Illumina, Inc., San Diego, CA, USA) with 500-ng RNA as input according to the manufacturer’s protocol. Libraries were sequenced on a MiSeq (Illumina, Inc., San Diego, CA, USA) with > 3 million reads per case, and sequences were analyzed using the RNA-Seq Alignment workflow, version 2.0.1 (Illumina, Inc., San Diego, CA, USA). The Integrative Genomics Viewer (IGV), version 2.2.13 (Broad Institute, REF), was used for data visualization [15]. In addition, DNA from cases 1, 2, 9, 10, and 20 was tested for mutations using the TruSight-Oncology500 Panel (Illumina, Inc., San Diego, CA, USA) according to the manufacturer’s protocol. The TruSight-Oncology500 Panel includes *ATRX*, *DAXX*, and *MEN1*, the three most frequently mutated genes in NETs.

### FISH testing for *EWSR1* rearrangements

In Case 1 and 9, fluorescence in situ hybridization (FISH) was performed on sections cut from formalin-fixed paraffin-embedded tissue blocks using the ZytoLight ® SPEC *EWSR1* Dual Color Break Apart Probe which is designed to detect translocations involving the chromosomal region 22q12.2 harboring the *EWSR1* gene (retrieved from ZytoVision, Bremerhaven, Germany) with standard protocols according to the manufacturer’s instructions.

## Results

### Clinical and demographic features

The major clinicopathological features are summarized in Table 1. The cohort included 12 females and 8 males (one of unspecified sex), aged 2 to 81 years (median, 49). The tumors originated in the pancreas (*n* = 10), lung (*n* = 9), thymus/mediastinum (*n* = 1), and kidney (*n* = 1).

**Table 1:** Clinicopathological and molecular features of ACTH-producing neuroendocrine neoplasms (*n* = 21)

No	Age/sex	Site	Histology	SSTR2A	BCOR IHC	RNA panel	Other molecular findings (TSO500 DNA panel)
1	53/M	Pancreas	NETG3 (Ki67:55%)	+	+ (wk.)	**EWSR1::BEND2** t(X;22)(p22;q12): chr22:29,688,158 (Exon 10; 3′-end) chrX:18,234,853 (Exon 2; 5′-end)	No pathogenic mutations in *MEN1, ATRX, DAXX, FUBP1, BCOR, VHL*, or other genes
2	29/M	Pancreas	NETG3 (Ki67:25%)	+	-	**KMT2A::BCOR** t(11;X)(q23;p11)**:** chr11:118,350,953 (Exon 6, 3′-end) chrX:39,923,852 BCOR (Exon 7, 5′-end)	Likely pathogenic *TSC1* mutation (p.S505LfsTer27; VAF: 3%) No pathogenic mutations in *MEN1, ATRX, DAXX, FUBP1, BCOR, VHL*, or other genes
3	NA	Pancreas	NETG2 (Ki67: 5%)	NA	-	Failed	No *BCOR* mutation*
4	34/F	Pancreas	NETG2 (Ki67:10%)	+	+ (wk.-moderate)	Failed	No *BCOR* mutation*
5	15/M	Pancreas	NETG2 (Ki67:4%)	+	NA	Failed	No *BCOR* mutation*
6	02/M	Pancreas	NETG1 (Ki67: < 2%)	NA	NA	Failed	No* BCOR* mutation*
7	63/F	Pancreas	NETG2 (Ki67:20%)	+	+ (wk.)	Failed	No *BCOR* mutation*
8	44/F	Pancreas	NETG2 (Ki67:3%)	+	-	Failed	No *BCOR* mutation*
9	36/F	Pancreas	NETG1 (Ki67: < 2%)	+	-	**EWSR1::BEND2** t(X;22)(p22;q12): chr22:29,688,595 (Exon 11; 3′-end) chrX:18,222,035 (Exon 5; 5′-end)	No pathogenic mutations in *MEN1, ATRX, DAXX, FUBP1, BCOR, VHL*, or other genes
10	48/F	Pancreas	NETG3 (> 20%)	+	NA	**TFG::ADGRG7** t(3;3) (q12.1; q12.1): Chr3: 100,438,902 (Exon 3; 3′-end) Chr3: 100,348,442 (Exon 2; 5′-end)	*FUBP1*: c.289dupA, p.S97Kfs*25 (53%). *VHL*: c.331A > T, p.S111C (60%); no *ATRX, DAXX, MEN1*, or *BCOR *mutations
11	21/M	Lung	AC (Ki67: 3%)	NA	-	Failed	No *BCOR* mutation*
12	49/F	Lung	TC (Ki67: 1%)	NA	-	No fusion (poor quality)	No *BCOR* mutation*
13	70/M	Lung	AC (Ki67: 24%)	-	NA	No fusion	No *BCOR* mutation*
14	48/F	Lung	AC (Ki67: 30%)	NA	+ (wk.)	No fusion (poor quality)	No *BCOR* mutation*
15	60/F	Lung	AC (Ki67: 2.4%)	+	NA	Failed	No *BCOR* mutation*
16	74/M	Lung	AC (Ki67: 2%)	-	-	Failed	No *BCOR* mutation*
17	49/F	Lung	AC (Ki67: 2%)	+	-	Failed	No *BCOR* mutation*
18	53/F	Lung	AC (Ki67: 5%)	+	-	No fusion	No *BCOR* mutation*
19	34/F	Lung	TC (Ki67: 1%)	+	-	No fusion	No *BCOR* mutation*
20	42/M	Mediastinal/thymus	AC (Ki67: 13%)	-	-	No fusion (poor quality)	No *ATRX, DAXX, MEN1*, or *BCOR *mutations
21	54/F	Kidney	AC (Ki67: 12%)	-	-	No fusion	No *BCOR* mutation*

*AC*, atypical carcinoid; *ANED*, alive with no evidence of disease; *DOD*, died of disease; *mo*, months; *NA*, not available; *NET*, neuroendocrine tumor; *NEC*, neuroendocrine carcinoma; *post-op*, postoperative; *TC*, typical carcinoid; *wk.*, weak

^*^Verified using the RNA-based variant assessment

### Histological and immunohistochemical findings

**Fig. 1:**
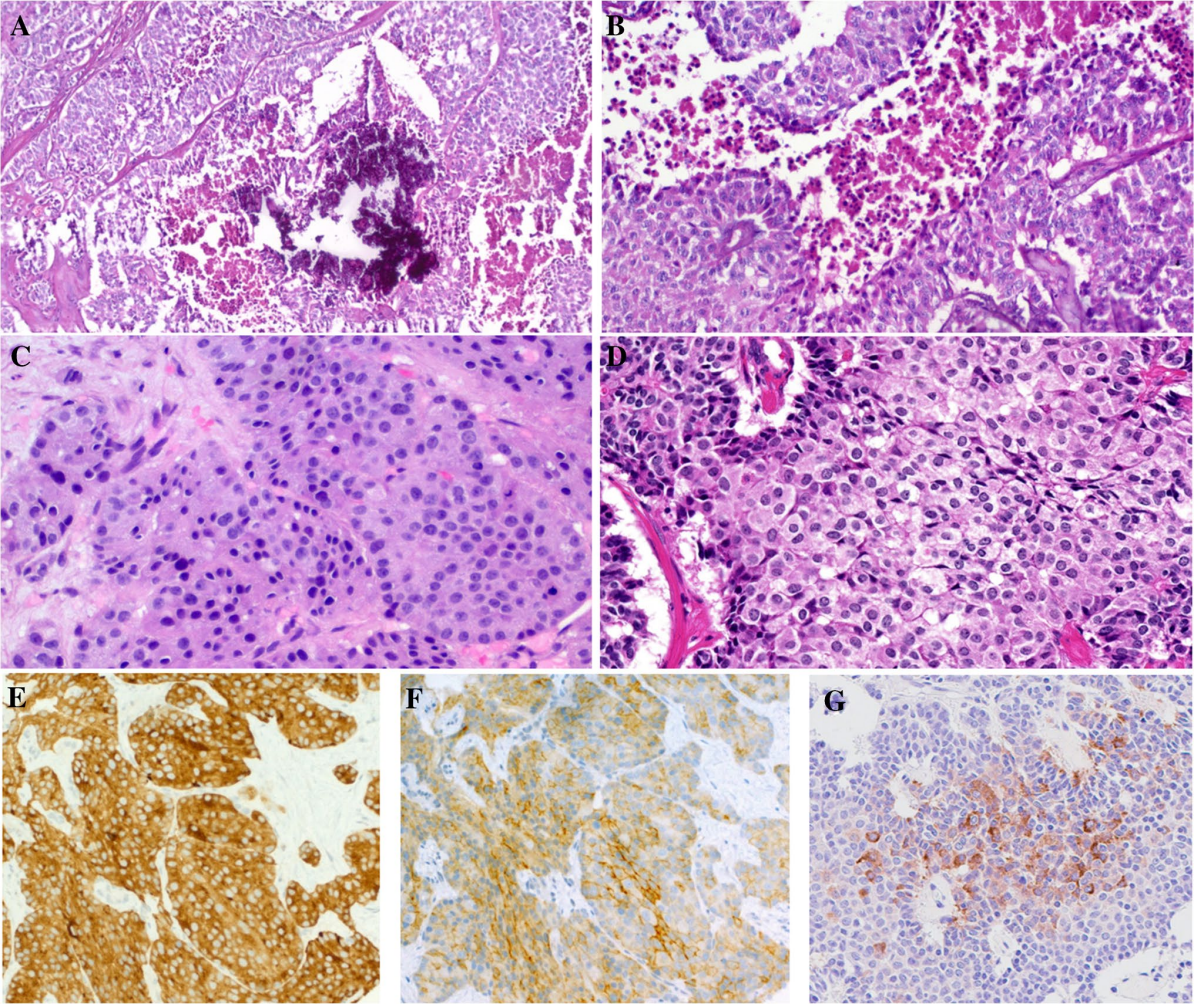
Representative examples of pancreatic ACTH-secreting neuroendocrine neoplasms. **A** + **B** This case (case 1 in Table 1 with *EWSR1::BEND2* fusion) showed features of NETG3 with extensive foci of necrosis. **C** Liver metastasis from pancreatic NETG3 with *KMT2A::BCOR* fusion (case 2). **D** Primary pancreatic NETG3 (case 10 in Table 1 with *TFG::ADGRG7* fusion). All tumors expressed synaptophysin (**E**), chromogranin-A (not shown), somatostatin receptor 2A (**F**), and ACTH (**G**)

**Fig. 2:**
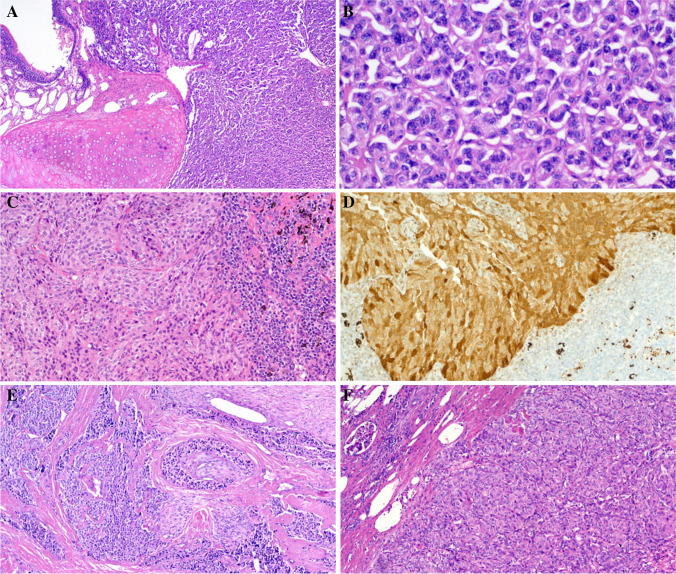
Representative examples of non-pancreatic ACTH-secreting neuroendocrine neoplasms. **A** + **B** Pulmonary typical carcinoid showing bronchocentric growth in **A**. **C** Pulmonary atypical carcinoid metastatic to peribronchial lymph node. **D** The same case shows strong diffuse ACTH expression in the nodal metastasis. **E** Thymic atypical carcinoid with extensive perineural invasion and stromal sclerosis. **F** Renal atypical carcinoid

The tumor tissue was obtained from resection specimens. This also applies to the cases seen in consultation. The pancreatic tumors were graded as G1 (2 cases), G2 (5 cases), and G3 (3 cases). Of the pulmonary NENs, 7 tumors qualified as atypical carcinoid and two as typical carcinoid. The kidney and the thymic tumors were both graded as atypical carcinoids, corresponding to NET G2. All tumors were purely neuroendocrine; none of the pancreatic and the non-pancreatic tumors had a non-neuroendocrine component. Histologically, the tumors showed mainly a solid growth pattern and were composed of tumor cells with round nuclei and rich eosinophilic cytoplasm (Figs. 1, 2). Unequivocal strong cytoplasmic expression of ACTH was detected by immunohistochemistry in all 21 cases.

The somatostatin receptor 2A showed a strong membranous expression in all pancreatic tumors, but only a weak and incomplete membranous reactivity in one of eight pulmonary tumors, and no expression in the mediastinal and renal tumors.

### Results of BCOR immunohistochemistry

**Fig. 3:**
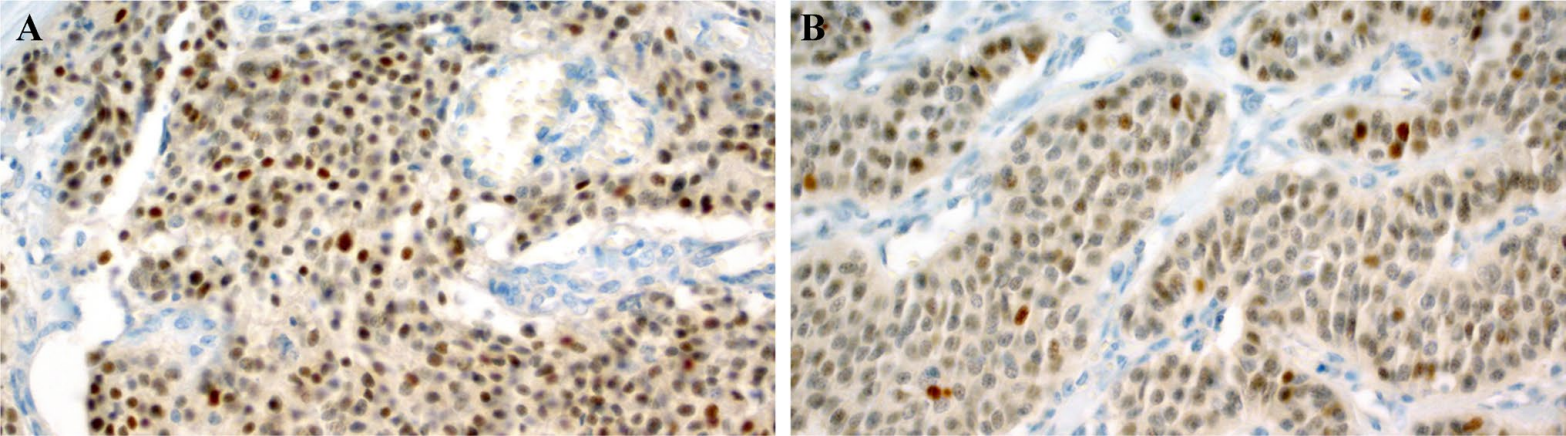
Representative examples of BCOR immunostaining. **A** This pancreatic tumor showed moderate expression in most of the tumor cell nuclei (case 4). **B** This pulmonary tumor revealed weak nuclear reactivity (case 14)

Overall, 16 cases revealed assessable results, with few cases showing suboptimal staining quality probably due to the old age of the tissue samples. BCOR expression was found in four tumors (25%), three of seven of pancreatic (Fig. 3A) and one of seven of pulmonary origin (Fig. 3B). Only one of three fusion-positive pancreatic NENs was BCOR-immunopositive. Notably, the BCOR-rearranged tumor showed no expression by immunohistochemistry.

### Molecular results

**Fig. 4:**
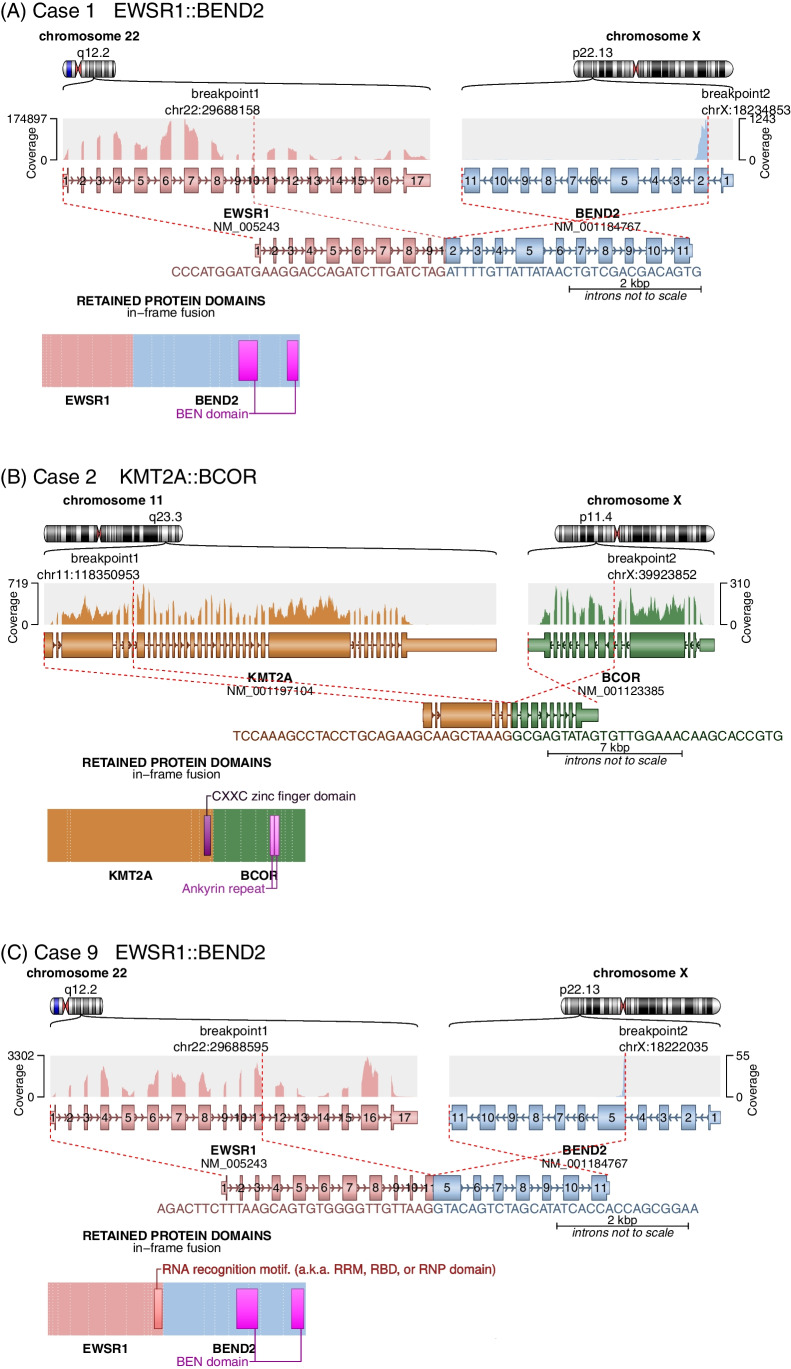
Schematic representation of gene fusions detected by panel sequencing. Illustrated is the gene structure of each fusion partner with the read coverage by panel sequencing, as well as the preserved exons resulting from the fusion event. The encoded chimeric protein including the retained protein domains is shown below. Depicted are gene fusions involving the genes *EWSR1* and *BEND2 *(A+B = cases 1 and 9), as well as the *KMT2A::BCOR* fusion detected in case 2 (C). Arriba_v2.3.0 with RefSeq annotation was employed for visualization purpose (Uhrig et al., 2021) [44]

Overall, targeted RNA sequencing was successful in a total of 11 cases: 4 of 10 pancreatic tumors, 5 of 9 pulmonary tumors, and in the one renal and one thymic tumor (Table 1). All four successfully tested pancreatic tumors revealed a gene fusion: two had a *EWSR1::BEND2* fusion and one case each had a *KMT2A::BCOR* and a *TFG::ADGRG7* fusion, respectively (Fig. 4). A critical review of the histology of the two *EWSR1*-fused pancreatic cases revealed no evidence of neuroectodermal or mesenchymal differentiation. *EWSR1* rearrangements were confirmed in both tumors using a *EWSR1* FISH probe.

All five cases tested with the TruSight Oncology500 (TSO500) gene panel (cases 1, 2, 9, 10, and 20) revealed no pathogenic or likely pathogenic mutations in *ATRX*, *DAXX* and *MEN1*, *BCOR*, or any of the other genes included in the panel. However, case 10 harbored pathogenic mutations in *FUBP1* and *vhl* in addition to the *TFG::ADGRG7* fusion. This patient has no sign or family history of von Hippel-Lindau syndrome. *BCOR* was assessed in the remaining 16 cases using RNA-based variant assessment; none showed evidence of a pathogenic or likely pathogenic *BCOR* mutation.

## Discussion

The genetic landscape of NENs has been emerging with several recently published comprehensive genetic studies. Inactivating somatic mutations in *ATRX*, *DAXX*, and *MEN1* represent the major mutations encountered in pancreatic NETs, but are much less frequent in their thoracopulmonary counterparts, indicating distinct site-dependent genetic pathways driving these tumors [11, 16]. A recent whole-genome study on 102 primary pancreatic NETs uncovered four major molecular pathways altered in them. The major affected genes are those involved in chromatin remodelling, DNA damage repair, mTOR signaling activation, and telomere maintenance [17]. On the other hand, the genetic background of ACTH-producing NENs remains poorly studied.

In this study, we detected gene fusions in all four successfully tested ACTH-producing pancreatic NETs with associated Cushing syndrome. Two of the four gene fusions were *EWSR1::BEND2* fusions which are exceptionally rare in solid cancers. They have been originally described in a subset of gliomas with astroblastoma-like morphology (eight cases reported to date) [18–20]. In these rare gliomas, *EWSR1* represents an alternate fusion partner, which however became more frequently encountered than the originally described *MN1::BEND2* fusion [18–20]. In decreasing order of frequency, *EWSR1*, *MN1*, and *MAMLD1* are the reported fusion partners of *BEND2* in astroblastoma-like gliomas [18–21]. It has been hypothesized that the fusion of MN1, EWSR1, or MAMLD1 with BEND2 enhances the expression of BEND2 downstream of the breakpoint and consequently drives oncogenesis [18–21]. Additional tumor types with *BEND2* fusions include single-case reports of unclassified basal cell-like salivary gland adenocarcinoma with a *EWSR1::BEND2* fusion [22] and one spindle cell soft tissue sarcoma with *MN1::BEND2* fusion [23].

In pancreatic NETs, *EWSR1::BEND2*-fusions have only been once reported. Two tumors among the 102 primary pancreatic NETs analyzed by Scarpa et al. in the above cited whole-genome sequencing study carried the *EWSR1::BEND2* fusion [17]. One of these reported *EWSR1::BEND2* fusion positive NETs was an ACTH-secreting tumor, while the other was a nonfunctioning NET (personal communication given by Prof. Aldo Scarpa, University of Verona). A third case in that same study harbored a *EWSR1::FLI1* fusion [17]. In addition, a *CDH7::BEND2* fusion has been found in a case report of a hepatic metastasis of a pancreatic NET G3, but there was no mention of ectopic ACTH secretion by the tumor [24]. All fusion-positive pancreatic NENs were reportedly typical NETs without evidence of mesenchymal elements, and they lacked membranous CD99 expression [17], ruling out the possibility of mesenchymal mimics with neuroendocrine-like features [25].

In the remaining two fusion positive cases of our study, we detected a *KMT2A::BCOR* and a *TFG::ADGRG7* fusion, respectively, but no fusion was detected in any of the 7 successfully analyzed ACTH-producing tumors of pulmonary (*n* = 5), renal (*n* = 1), or thymic (*n* = 1) origin. Although limited by the low number of successfully tested cases, our results indicate a potentially high frequency of recurrent gene fusions (mainly *EWSR1::BEND2*) in pancreatic ACTH-producing NENs. Inclusion of different tumor grades in our cohort argues against the possibility of a grade-dependent fusion association. Taken together (including the personal communication with Prof. Scarpa), three of the four pancreatic NETs with an *EWSR1::BEND2* fusion were ACTH-producing, suggesting a significant association between this gene fusion and the ectopic ACTH production. Notably, the significant enrichment of our pancreatic ACTH-producing NETs for the *EWSR1::BEND2* fusion (found in 2 of 4 successfully tested cases = 50%) strikingly contrasts with the very low overall frequency of this fusion in unselected pancreatic NETs (2% [17]).

The *KMT2A::BCOR* fusion which we detected in one tumor is novel. Although it is not clear, if the two different fusions *EWSR1::BEND2* and *KMT2A::BCOR* might have functional relationship and hence represent two mutually exclusive alternate molecular pathomechanisms in ACTH-producing pancreatic NETs, it is noteworthy that two of the reported *EWSR1::BEND2*-rearranged astroblastoma-like gliomas expressed BCOR by immunohistochemistry, despite the absence of molecular *BCOR* alterations [20]. This suggests some oncogenic interactions between BEND2 and BCOR. Moreover, a recently reported case of ACTH-secreting pulmonary carcinoid revealed a *BCOR* mutation, representing another argument for a potential role of BCOR in the pathogenesis of ACTH-producing NENs lacking gene fusions [14]. The *KTM2A::BCOR* fusion we detected herein has been only recently reported in a single case of synovial chondromatosis [26], but it represents a novel fusion in the context of a NEN. Using RNA-based variant assessment, we detected no pathogenic *BCOR* mutations in our fusion-negative cases, but the RNA-based method is of limited reliability and direct DNA-based sequencing of *BCOR* is mandatory to rule out *BCOR* mutations. BCOR immunohistochemical analysis as surrogate for BCOR alterations revealed no significant overexpression across the different molecular subgroups of the NETs we tested, indicating the limited value of BCOR IHC in this context. The *TFG::ADGRG7* fusion detected herein in our fourth fusion-positive case remains of unknown significance. TFG is a well-known fusion partner in a variety of mesenchymal and epithelial neoplasms of different organs, mostly fused to other receptor tyrosine kinases such as ALK, ROS1, and NTRK [27]. However, in a recent study, the *TFG::ADGRG7* fusion was detected in a T-cell lymphoblastic leukemia sample and in normal tissue so it remains unclear, if it was present in the germline and, if yes, whether it is pathogenic or it represents a benign variant [28]. This remains an issue of future studies.

In light of the recurrent nature of the *EWSR1::BEND2* fusions (detected in two of our cases and in the one ACTH-positive tumor in the Scarpa et al. study [17]), it seems that these fusions are likely driver events in these tumors as they proved to be mutually exclusive with *ATRX*, *DAXX*, and *MEN1* mutations (as the most frequently mutated genes in NETs) in all four fusion-positive cases.

The occurrence of these unusual gene fusions in 3 of 4 ACTH-secreting pancreatic NENs makes us think that these findings are not a mere coincidence, but might be, in one way or another, related to the triggering of ectopic hormone production. It is well known that several mesenchymal entities driven by gene fusions including *STAT6:NAB2* (hypoglycemia in 5% of solitary fibrous tumors), *ETV6::NTRK3* (neonatal hypercalcemia in infantile fibrosarcoma/congenital mesoblastic nephroma), and *EWSR1::CREB1* (hyper-Interleukin-6 in angiomatoid fibrous histiocytoma) are specifically associated with paraneoplastic phenomena [29]. Excessive production of IL-6 by the neoplastic cells of angiomatoid fibrous histiocytoma represents the major factor responsible for its associated paraneoplastic symptoms [30]. Notably, the presence of a CREB-binding site in the IL-6 promotor region together with the high expression of CREB triggered by the *EWSR1::CREB1* fusion has been proposed to explain the IL-6 overexpression observed in this rare tumor type [30–32]. Moreover, paraneoplastic hypercalcemia, while being specifically associated with the *ETV6::NTRK3* fusions, is also significantly associated with *SMARCA4* inactivation in the majority of small cell carcinoma of the ovary, hypercalcemic type [29]. The same principle might apply to the observations that ectopic ACTH production can be associated with a variety of molecular genetic alterations including *EWSR1::BEND2* and other gene fusions as in our cases, *BCOR* mutation as in one reported typical pulmonary carcinoid [14], and *EWSR1::ETS* fusions as in two recently reported Ewing sarcoma cases [33, 34].

ACTH-producing NENs express pro-opiomelanocortin (POMC), the precursor of ACTH, in addition to other pre-ACTH precursor molecules [1]. The gene encoding POMC is mapped to 2p23.3. The regulation of POMC expression seems highly complex and underlies different mechanisms, under both physiological and pathological conditions [35–42]. Hypomethylation of the *POMC* promoter, demethylation of the E2F1-binding site, and others have been suggested as a possible explanation for the ectopic secretion of POMC by non-corticotroph tumor cells [39, 42, 43]. In this study, we suggest possible additional mechanisms of overexpression of ACTH-precursors and hence ectopic ACTH production by the neoplastic cells of NEN via a yet unknown mechanism that is likely related to the underlying gene fusion, at least in a subset of cases. In this regard, molecular/structural homology between parts of the fusion transcript and the promoter of POMC or other pre-ACTH precursors might be responsible for the overexpression of the ACTH precursors, but this remains currently speculative and merits functional verification in future studies.

In summary, we herein have reported gene fusions involving *BEND2*, *BCOR* and *TFG* in four successfully tested ACTH-producing and Cushing syndrome-associated NENs of the pancreas, but not in those from non-pancreatic origin. This unusual genotype might be involved in the triggering of ectopic ACTH hormone production in pancreatic NENs, the mechanisms of which deserve future functional and genetic in-depth studies.

## Data Availability

The data that support the findings of this study are available on request from the corresponding author. The data are not publicly available due to privacy or ethical restrictions.
